# Human Papillomavirus Type-Specific Prevalence in the Cervical Cancer Screening Population of Czech Women

**DOI:** 10.1371/journal.pone.0079156

**Published:** 2013-11-12

**Authors:** Ruth Tachezy, Jana Smahelova, Jana Kaspirkova, Martina Salakova

**Affiliations:** 1 Department of Experimental Virology, Institute of Hematology and Blood Transfusion, Prague, Czech Republic; 2 Bioptic Laboratory, Pilsen, Czech Republic; Universidad Nacional de La Plata., Argentina

## Abstract

**Background:**

Infection with high-risk human papillomavirus (HPV)types has been recognized as a causal factor for the development of cervical cancer and a number of other malignancies. Today, vaccines against HPV, highly effective in the prevention of persistent infection and precancerous lesions, are available for the routine clinical practice.

**Objectives:**

The data on the prevalence and type-specific HPV distribution in the population of each country are crucial for the surveillance of HPV type-specific prevalence at the onset of vaccination against HPV.

**Methods:**

Women attending a preventive gynecological examination who had no history of abnormal cytological finding and/or surgery for cervical lesions were enrolled. All samples were tested for the presence of HPV by High-Risk Hybrid Capture 2 (HR HC2) and by a modified PCR-reverse line blot assay with broad spectrum primers (BS-RLB).

**Results:**

Cervical smears of 1393 women were analyzed. In 6.5% of women, atypical cytological findings were detected. Altogether, 28.3% (394/1393) of women were positive for any HPV type by BS-RLB, 18.2% (254/1393) by HR HC2, and 22.3% (310/1393) by BS-RLB for HR HPV types. In women with atypical findings the prevalence for HR and any HPV types were significantly higher than in women with normal cytological findings. Overall, 36 different HPV types were detected, with HPV 16 being the most prevalent (4.8%). HPV positivity decreased with age; the highest prevalence was 31.5% in the age group 21-25 years.

**Conclusions:**

Our study subjects represent the real screening population. HPV prevalence in this population in the Czech Republic is higher than in other countries of Eastern Europe. Also the spectrum of the most prevalent HPV types differs from those reported by others but HPV 16 is, concordantly, the most prevalent type. Country-specific HPV type-specific prevalences provide baseline information which will enable to measure the impact of HPV vaccination in the future.

## Introduction

Human papillomaviruses (HPVs) are small DNA viruses which infect mucosal and skin epithelia. Currently more than 170 HPV types are known, 13 classified as high-risk and 7 as probably high-risk HPV types. The low risk (LR) HPV types cause benign lesions while high risk (HR) HPV types have been established as etiological agents of cervical cancer (CC) [1,2]. Besides cervical cancer, HR HPV has carcinogenic effects at several other anatomical sites in women and men [3]. HPVs are currently the most common viral sexually transmitted infection worldwide but the distribution of different genotypes varies across populations and geographical regions [4]. In a meta-analysis of 1 million women with normal cytological findings, the estimated global HPV prevalence was 11.7% and there were substantial differences between continents. However, five HPV types were consistently the most prevalent: HPV 16, 18, 52, 31, and 58, regardless of the geographical location. In Europe, the highest prevalence was found in Eastern countries [5]. The data from the Czech Republic published before [6] were not included in the meta-analysis because the study did not meet the inclusion criteria due to an inappropriate selection of women with normal cytological findings, since they were recruited in a hospital based setting. The prevalence of HPV in this group was high (23%) but similar to other Eastern countries. High HPV prevalence was reported to correlate with high incidence of CC with two exceptions, Southern Asia and Eastern Europe [5]. However, in the Czech Republic the incidence of CC is consistently very high, 16.1/100 000 women (European standard) in 2010, despite the fact that considerable financial resources are directed toward cervical cancer prevention. The reasons are mainly a lack of coordination and monitoring that leads to the over-screening of a minority of women while the majority of the target population are under-screened or not screened at all [7]. The age recommended by the Czech Gynecological and Obstetrical Society for the first examination of women after the onset of sexual life is 15 years and there is no limitation in terms of screening age specified in the program for cervical cancer prevention in the Czech Republic. However, women aged 25 to 60 who did not attend the examination in the last two years should be invited by the health insurance company [8].

HPV detection has been shown as a robust, reproducible, and sensitive test for triage of women with abnormal cytological findings and for follow-up of women treated for cervical lesions. Furthermore, HPV detection has also been recently shown to be a sensitive and cost-effective test for the identification of women at risk in primary cervical screening programs [9,10]. Two prophylactic papillomavirus vaccines, bivalent and quadrivalent, are available for primary prevention of HPV-associated diseases [11]. The evaluation of HPV type-specific prevalence in the population before the onset of routine vaccination and the surveillance of possible changes in type distribution is important both for the selection of the diagnostic tests and for the estimation of the potential local impact of HPV vaccines on the prevention of HPV-associated diseases in women and men. 

## Materials and Methods

### Study population

In the Czech Republic the screening interval is one year. Women with atypical cytological and/or colposcopical findings are scheduled for follow up examination in 3-6 months interval. HPV detection and typing was performed in women who had their previous gynecological examination more than one year ago, and who had no history of atypical or pathological findings on the cervix uteri and no previous treatment for cervical lesions.

Altogether, samples of 1393 women (mean age 33.5 years; age range 14 - 79 years) were selected from the bank of samples of the Bioptic Laboratory in Pilsen, Czech Republic. These samples were provided by gynaecologists who treat patients in 5 of 14 different districts in the Czech Republic in the period between January 2010 and December 2011. These locations include both big cities and rural areas. Samples in Sample Transport Medium (STM) (Qiagen, Hilden, Germany) were sent to the National Reference Laboratory for Papillomaviruses (NRL PV) under the laboratory identification number (lab ID) to preclude the personal identification of study subjects in NRL PV. The study was double blind. The people performing HPV DNA detection and typing were not aware of the cytology results and the cytologists were blinded to HPV DNA results. The cytological results were available under the lab ID for the final analysis. 

#### Cytological classification

The cytological classification was done according to Bethesda 2001 update [12]. For the purpose of this study, the cytological findings were grouped into four categories: normal, minor (if atypical squamous cells of unknown significance /ASC-US/ and/or atypical glandular cells, not otherwise specified /AGC-NOS/ were detected), mild (if low-grade squamous intraepithelial lesions /LSIL/ were detected), or severe (if atypical squamous cells, cannot exclude high-grade squamous intraepithelial lesion /ASC-H/ and/or atypical glandular cells, favour neoplasia /AGC-NEO/, and/or high-grade squamous intraepithelial lesions /HSIL/) were detected). 

#### Ethic statement

The study was approved by the ethic committee of the Institute of Hematology and Blood Transfusion. The need for consent form was waived by the ethics committee because the samples were selected anonymously from the bank of samples of the Bioptic Laboratory in Pilsen, Czech Republic (http://www.biopticka.cz/).

#### Data availability

The study source data will be made available upon request, in accordance with the principles established by the US National Research Council of the National Academies (National Academies Press, 2003).

### HPV detection and genotyping

#### Hybrid Capture 2 assay (HC2)

A 200 µl aliquot from each Sample Transport Medium (STM) (Qiagen, Hilden, Germany) specimen was stored at -20°C until further processed (see below). All specimens were tested using the Hybrid Capture® 2 High-Risk HPV DNA Test (HR HC2) (Digene Corporation, Gaithersburg, MD). This test allows for the detection of 13 HR HPV types (HPV 16, 18, 31, 33, 35, 39, 45, 51, 52, 56, 58, 59, and 68). Testing was performed according to the instruction of the manufacturer. The threshold for positive HPV designation was a relative light unit/cutoff (RLU/CO) ratio of ≥1.0. 

#### PCR and hybridization

Cells from a 200 µl aliquot of each STM specimen were pelleted by centrifugation and DNA was extracted using the QIAamp DNA mini kit (Qiagen, Hilden, Germany) according to the manufacturer’s protocol. The final elution of DNA from the column was done by 100 µl of the elution buffer. 

The presence and integrity of the human DNA were confirmed by PCR with the primers MS3/MS10bio specific for the beta-globin gene [13]. 

The amplification was performed in a PCR thermocycler (PTC 200, MJ Research Inc., Waltham, MA, USA) with a mixture of broad spectrum BSGP5+ primers and 5′-end biotin labelled BSGP6+ primers which amplify the 150 bp fragment of the L1 gene [13]. Fifty microlitres of the PCR mixture contained 4 µl of the isolated DNA, 50 mM KCl, 10 mM Tris HCl, pH 8.3 (Roche Applied Biosystems, Mannheim, Germany), 200 µM of each deoxynucleoside triphosphate, 3.5 mM MgCl2, 1 U of DNA AmpliTaq Gold polymerase (Roche Applied Biosystems, Mannheim, Germany), 10 pmol of each of the nine BSGP5+ primers, and 20 pmol of each of the three 5‘-biotinylated BSGP6+ primers (MWG-Biotech AG, Ebersberg, Germany). After initial denaturation at 94°C for 4 minutes, each of the 40 cycles consisted of denaturation at 94°C for 20 seconds, primer annealing at 38°C for 30 seconds, and chain elongation at 71°C for 80 seconds. At the end, incubation at 71°C for 4 minutes was performed. After amplification, 10 µl of the PCR product was analyzed on a horizontal 3% agarose gel (NuSieve 3:1 agarose, FMC BioProducts, Rockland, ME). 

For genotyping, the reverse line blot hybridization (RLB) was used [14]. The RLB method is able to identify 37 different HPV types in a single assay. Sequences of all probes, except for those redesigned according to Schmitt [15] (HPV types 16, 39, 59, and 82, and universal probes U1 and U2), were described before [14]. All probes were covalently linked with the 5′-terminal amino-group to an activated negatively charged Biodyne C membrane and hybridized with the biotinylated PCR product. After washing, the membrane was incubated with peroxide-labeled streptavidin conjugate at 42°C for 60 min. For chemiluminiscent detection of hybridizing DNA, the membrane was incubated in ECL detection liquid (Amersham Biosciences, Uppsala, Sweden) and exposed to LumiFilm (Roche, Indianapolis, IN) for 5 min. 

The samples positive by HR HC2 but negative by BS-RLB were tested by PCR with the consensus primers MY09/MY11 and HMB01 as specified before [16]. Positive samples were typed by sequencing as shown below. 

#### HPV sequencing

The samples that had not hybridized on the RLB but had revealed a clear band on the agarose gel were subjected to DNA nucleotide sequencing in order to determine the exact HPV genotype. Forty microlitres of the PCR product was cut out from the 2% agarose gel (NuSieve GTG agarose, FMC BioProducts, Rockland, ME, USA), purified using the MinElute Gel Extraction kit (Qiagene, Hilden, Germany), and sequenced with the “BigDye Terminator Primer Cycle Sequencing kit” (Applied Biosystems, Foster City, CA, USA). The analysis was performed on an automated ABI PRISM 3500 sequencing system (Applied Biosystems, Foster City, CA, USA) and the sequences were analysed by the Chromas software and evaluated by the BLAST software (http://www.ncbi.nlm.nih.gov/BLAST/). 

#### Linear Array HPV genotyping test (LA)

Samples with discrepant findings by the HR HC2 and PCR methods (see above) were tested with the LA (Roche). This test allows for the detection of 37 HR and LR HPV types (HPV 6, 11, 16, 18, 26, 31, 33, 35, 39, 40, 42, 45, 51, 52, 53, 54, 55, 56, 58, 59, 61, 62, 64, 66, 67, 68, 69, 70, 71, 72, 73 (MM9), 81, 82 (MM4), 83 (MM7), 84 (MM8), IS39, and CP6108). The testing was performed according to the instruction of the manufacturer. From the extracted DNA, the same amount as for the BS-RLB method (4 µl) was used. 

### Statistical analyses

Multiply infected samples were those in which two or more HPV types had been detected. Such samples were counted as positive for one type of HPV and also included among positives for the others. The type-specific HPV prevalences are expressed as percentages of all subjects tested for HPV, and thus represent the HPV prevalence in either single or multiple infections. To express the representation of each type in single and multiple infections, each sample was assigned in proportional fractions to particular genotypes but counted only once [17]. The difference in the HPV prevalence in the group of women with normal cytological findings were compared to those women with atypical findings (ASC-US/AGC-NOS, AGC-NEO/LSIL, ASC-H/HSIL) with 95% confidence intervals (CI) using GraphPad InStat (version 3.00) (GraphPad Software, San Diego, CA). For contingency tables, the standard chi-square test and the Fisher exact test were used. All tests were two sided and the significance level was a=0.05. In order to exclude the differences in age structure between populations we used direct standardization (WHO World standard). The Kappa statistic was used to measure the agreement for HPV positivity status between the two tests used. 

The detected HPV types were classified into low-risk (LR) (HPV 6, 11, 32, 40, 42, 43, 44, 54, 61, 62, 72, 74, 81, 90, and 114), high-risk (HR) (HPV 16, 18, 31, 33, 35, 39, 45, 51, 52, 56, 58, 59, and 68), and probably high-risk (pHR) (HPV 26, 53, 66, 67, 70, 73, and 82) types of the genus Alpha that contains the mucosal types of HPV [18–20]. In our analyses, we defined (based on the phylogenetic relatedness) HPV 31 as closely related to HPV 16, and HPV 45 as closely related to HPV 18. HPV 33, 52, and 58 as more distantly related to HPV 16.

The laboratory is accredited to ČSN EN ISO 15 189 and participates regularly in external quality control programs organized by INSTAND (Germany) and Mendel Center for Biomedical Sciences (Cyprus). Furthermore, the laboratory participated twice in the WHO HPV LabNet Proficiency Study of HPV DNA Typing organized by the WHO HPV Global Reference Laboratory [21,22]. 

## Results

Out of 1393 women in our study, 25.6% (357/1393) were younger than 25 years and 2.2% were older (32/1393) than 60 years, i.e. 72% were within the recommended age range for screening. The majority of the women - 93.5% (1302/1393) - had normal cytological findings. Altogether, 6.5% (91/1393) of the women had abnormal cytological findings, 4.4% (61/1393) had ASC-US or AGC-NOS, 2.0% (28/1393) had AGC-NEO or LSIL and 0.1% (2/1393) had ASC-H or HSIL. Fewer women - 18.2% (254/1393) - were positive for HR HPV by the HR HC2 method in comparison to BS-RLB which detected 22.3% (310/1393) HR HPV or HR and LR HPV positive women and 28.3% (394/1393) were positive by BS-RLB for any HPV type. The corresponding prevalences, age-standardized to the world population, were 14.7%, 19.5%, and 24.6%, respectively. By BS-RLB, 70.6% (278/394) of subjects were HPV positive for a single HPV type while 29.4% were positive for multiple types (18.3% for two, 6.9% for three, 2.8% for four, 1.3% for five, and 0.3% for six HPV types). LR HPV types, as a single infection, were present in 6.0% (84/1396) of women (Table 1). 

**Table 1:** HPV prevalence by cytological findings and by HPV DNA detection method.

		CYTOLOGICAL FINDINGS				
	All	normal	ASC-US/ AGC-NOS	AGC-NEO/ LSIL	ASC-H/ HSIL	p-value^&^
	N [%]	N [%]	N [%]	N [%]	N [%]	
HR HC2	254 (18.2)	203 (15.6)	23 (37.7)	27 (96.4)	1 (50.0)	<0.0001
PCR, HR HPV*	310 (22.3)	256 (19.7)	27 (44.3)	26 (92.9)	1 (50.0)	<0.0001
PCR, any HPV	394 (28.3)	333 (25.6)	33 (54.1)	27 (96.4)	1 (50.0)	<0.0001
PCR, LR HPV**	84 (6.0)	77 (5.9)	6 (9.8)	1 (3.6)	0 (0.0)	0.4912
HPV 6/11^***^	20 (1.4)	19 (1.5)	0 (0.0)	1 (3.6)	0 (0.0)	0.7503
HPV 16/18^#^	104 (7.5)	86 (6.6)	9 (14.8)	9 (32.1)	0 (0.0)	<0.0001
HPV 31/45^***^	43 (3.1)	37 (2.8)	3 (4.9)	3 (10.7)	0 (0.0)	0.0565
HPV 33/52/58^&&^	48 (3.4)	38 (2.9)	6 (9.8)	3 (10.7)	1 (50.0)	0.0007

^&^ p-value for comparison of HPV prevalence in patient with normal and atypical (ASC-US/AGC-NOS, AGC-NEO/LSIL, ASC-H/HSIL) cytological findings, HR HC2= Hybrid Capture HR test, PCR= polymerase chain reaction, ASC-US= atypical squamous cells of unknown significance, AGC-NOS= atypical glandular cells, not otherwise specified, AGC-NEO= atypical glandular cells, favour neoplasia, LSIL= low-grade squamous intraepithelial lesion, ASC-H= atypical squamous cells, cannot exclude high-grade squamous intraepithelial lesion (HSIL), HSIL= high-grade squamous intraepithelial lesion

* presence of HR HPV and also HR HPV in multiple infection with LR HPV types, ** presence of LR HPV types only, in single or multiple infections with other LR types

*** samples which do not contain HPV 16 and/or 18, ^#^ samples HPV 16 and/or 18 positive, ^&&^ samples which do not contain HPV 16 and/or 18 and/or 31 and/or 45

Cytologically normal women were positive for HR HPV types in 15.6% (203/1302) by the HC2 test. Using BS-RLB, 19.7% (256/1302) of the cytologically normal women were positive for HR HPV types, and 25.6% (333/1302) for any HPV type (Table 1). The respective prevalences, age-standardized to the world population, were 12.5%, 16.3%, and 21.4%. Of the study women, 5.9% (77/1393) were infected with LR HPV types. 

**Figure 1:**
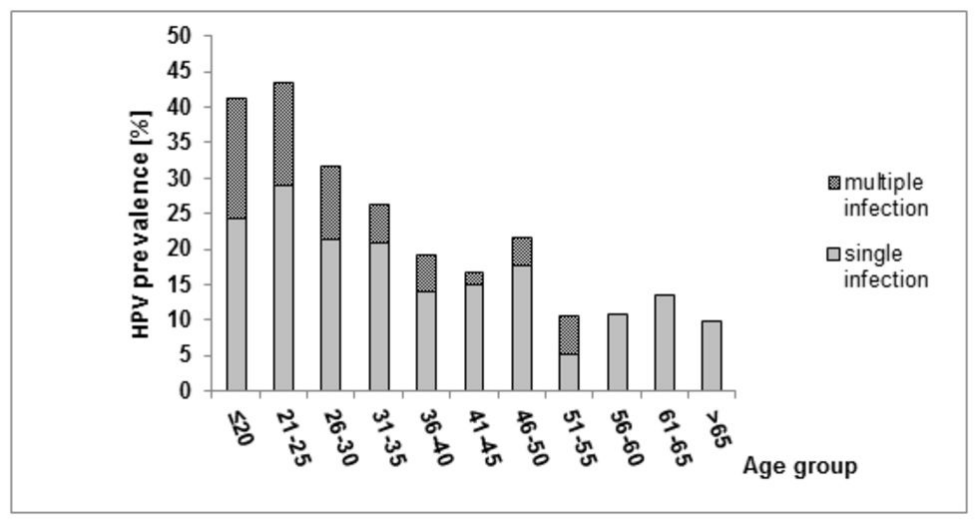
Frequency of single and multiple HPV infection in the screening population by age as revealed by BS-RLB method.

The prevalence of multiple infection increased with the severity of cytological findings (P for trend=0.0003) (Table 2). Multiple infection was more common in the younger age and was absent in women older than 55 years (Figure 1). 

**Table 2:** Age specific HPV prevalence by method of HPV DNA detection in women with normal cytological findings.

		HPV DNA PREVALENCE (%)					
Age group	N	HR HC2	PCR HR HPV	PCR ANY HPV	PCR LR HPV	HPV 6/11	HPV 16/18
≤20	153	34 (22.2)	43 (28.1)	54 (35.3)	11 (7.2)	4 (1.3)	13 (9.2)
21-25	213	67 (31.5)	73 (34.3)	87 (40.8)	14 (6.6)	7 (3.3)	26 (12.2)
26-30	202	29 (14.4)	43 (21.3)	56 (27.7)	13 (6.4)	4 (2.0)	18 (8.9)
31-35	216	34 (15.7)	42 (19.4)	54 (25.0)	12 (5.6)	3 (1.4)	12 (5.6)
36-40	192	18 (9.4)	24 (12.5)	36 (18.8)	12 (6.3)	2 (1.0)	6 (3.1)
41-45	114	10 (8.8)	13 (11.4)	17 (14.9)	4 (3.5)	0 (0.0)	3 (2.6)
46-50	90	5 (5.6)	9 (10.0)	17 (18.9)	8 (8.9)	0 (0.0)	7 (7.8)
51-55	55	5 (9.1)	4 (7.3)	5 (9.1)	1 (1.8)	0 (0.0)	3 (3.6)
56-60	37	1 (2.7)	3 (8.1)	4 (10.8)	1 (2.7)	0 (0.0)	0 (0.0)
61-65	21	0 (0.0)	2 (9.5)	3 (14.3)	1 (4.8)	1 (4.8)	0 (0.0)
>65	9	0 (0.0)	0 (0.0)	0 (0.0)	0 (0.0)	0 (0.0)	0 (0.0)

Regardless of the method used for HPV detection, the prevalence of HR HPV increased with the severity of cytological findings while the prevalence of LR HPV types in single or multiple infection remains more or less stable (Table 1). In our study, only two women were detected with severe cytological findings, one with HSIL was positive by both HR HC2 and BS-RLB (HPV 33) and one patient with ASC-H was negative by both methods. 

**Figure 2:**
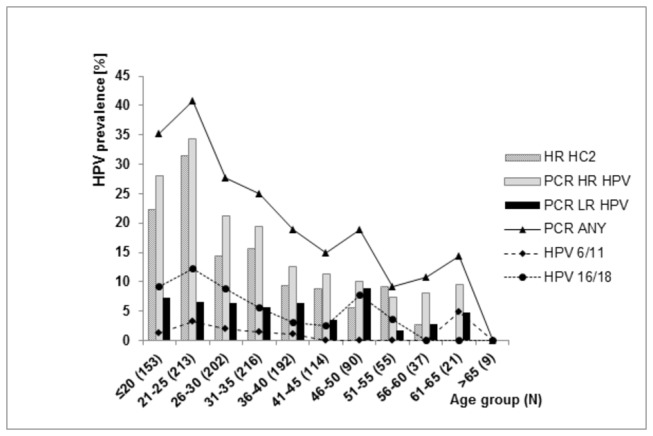
HPV prevalence by age and method of HPV DNA detection (PCR=BS-RLB, HPV6/11 and HPV 16/18 were detected by means of BS-RLB).

The prevalence of HR HPV was age dependent. The highest prevalence of HR HPV was observed in the age group 21-25 years, by both HR HC2 and BS-RLB, 34.2% (78/228) and 36.8% (84/228), respectively, and rapidly decreased in older women. In the age category of 61-65 years, BS-RLB detected 9.1% (2/22) of HR HPV positives and HR HC2 gave negative results (Figure 2). 

In women with normal cytological findings, the prevalence of HR HPV was slightly lower in comparison to the whole cohort, reaching the maximum of 31.5% (67/213) and 34.3% (73/213) at the age of 21-25 years, as assessed by HR HC2 and BS-RLB, respectively (Table 2). 

The detection of HR HPV DNA by HR HC2 and BS-RLB yielded concordant results in 91.4% (1273/1393) of samples. The correlation was substantial (Kappa value = 0.7339). 

Out of 120 discrepant samples, 88 were BS-RLB positive but HR HC2 negative for HR HPV. Out of these, 76.1% (67/88) contained HR HPV types which are targeted by the HR HC2 set. In 32.8% (22/67) of these samples, multiple HPV infection was detected. The vaccinal HR HPV types 16 and/or 18 were detected in 31.8% (28/88) of these samples: in 60.7% (17/28) as a single infection and in 39.3% (11/28) as part of a multiple infection. Three of these 28 patients (10.7%) with multiple infection had suspicious cytological findings (ASC-US) while the remaining 89.3% of subjects had normal cytology. 

There were 32 samples positive on HR HC2 but negative for HR HPV by BS-RLB. In 56.3% (18/32) of these samples, BS-RLB detected LR HPV types only (HPV 6, 32, 42, 43, 44, 54, 61, 62, 74, and 81); the remaining 43.8% (14/32) of samples were BS-RLB negative for any HPV type. The mean value of RLU/CO in those 14 samples was rather low (12.3 RLU/CO) which equals borderline to weak positivity of sample on HR HC2. In 10/18 samples, positive by BS-RLB for LR HPV types only, LA also detected HR HPV types, mostly as part of multiple infection. In 6/14 samples negative by BS-RLB for any type, LA also detected HR HPV types (Table 3). 

**Table 3:** Results of HPV type-specific detection by means of Linear Array (LA) in samples negative for HR HPV on BS-RLB but positive on HR HC2.

			HPV TYPE	
SAMPLE	CYTOLOGICAL FINDINGS	HR HC2 [RLU/CO]	BS-RLB	LA
1	Normal	8.14	42	42,66
2	Normal	1.23	42	42
3	ASC-US	14.33	42	42,51
4	Normal	3.2	0	0
5	Normal	1.7	42	42
6	LSIL	35.9	44,81*	31,62,81,52**
7	Normal	1.2	0	0
8	Normal	14.3	44	35*
9	Normal	132.7	0	0
10	Normal	1.6	0	0
11	Normal	1.1	0	0
12	ASC-US	2.5	42	42
13	Normal	11.96	0	0
14	Normal	6.2	74	0**
15	Normal	1.98	42	42,66,84
16	Normal	2.27	61	61
17	Normal	4.7	0	0
18	Normal	1.8	0	35,59
19	Normal	5.90	0	31
20	Normal	1.43	44,54*	0**
21	Normal	6.17	54*	31,54
22	Normal	1.47	0*	54,62
23	Normal	1.46	42	59
24***	Normal	5.22	62	31
25	Normal	1.21	32	83,84**
26	Normal	1.87	0	35
27	Normal	10.12	61	61,81
28	Normal	1.14	0	52
29	Normal	5.24	43	59**
30	Normal	3.90	0	52
31	Normal	1.50	0	59,81
32	Normal	23.77	6	6,31

* BS-RLB does not detect HPV 62, ** LA does not detect HPV 32, 43, 44, 74; *** HPV 66 detected by means of MY09/11 PCR and sequencing

Altogether, 35 different HPV types were detected: 20 HR and pHR HPV types and 15 LR HPV types. The prevalence by HPV type and cytological finding are shown in Table 4. The most prevalent HR types were HPV 16 (5.4%), followed by HPV 56, 39, 53, and 18, while of LR types, HPV 42, 54, 44, 6, and 61 were most frequently detected. The prevalences of vaccinal, closely and distantly related HPV types in all women as well as in women with normal cytology were almost identical and are summarized in Table 1. HPV 6 and/or 11 was only found in 1.4% (20/1393) of samples while HPV 16 and/or 18 in 7.5% (104/1393) of samples. Closely related types HPV 31 and/or 45 were present in 3.1% (42/1393) and distantly related types HPV 33 and/or 52 and/or 58 in 3.4% (48/1393) of samples. 

**Table 4:** The prevalence of HPV types in the screening population of Czech women by cytological findings as revealed by BS-RLB.

	CYTOLOGICAL FINDINGS									
TYPES	ALL		NORMAL		ASC-US/ AGC-NOS		AGC-NEO/ LSIL		ASC-H/ HSIL	
	N	%	N	%	N	%	N	%	N	%
	1393	100.0	1302	93.5	61	4.4	28	2.0	2	0.1
	N+	%	N+	%	N+	%	N+	%	N+	%
HPV ALL	394	28.3	333	25.6	33	54.1	27	96.4	1	50.0
HR HPV	310	22.3	256	19.7	27	44.3	26	92.9	1	50.0
HPV single	278	70.6	247	74.2	17	51.5	13	48.1	1	100.0
HPV multiple	116	29.4	86	25.8	16	48.5	14	51.9	0	0.0
**6**	20	1.4	19	1.5	0	0.0	1	3.6	0	0.0
**11**	2	0.1	2	0.2	0	0.0	0	0.0	0	0.0
**16**	75	5.4	63	4.8	7	11.5	5	17.9	0	0.0
**18**	32	2.3	24	1.8	4	6.6	4	14.3	0	0.0
**26**	1	0.1	1	0.1	0	0.0	0	0.0	0	0.0
**31**	22	1.6	16	1.2	3	4.9	3	10.7	0	0.0
**32**	1	0.1	1	0.1	0	0.0	0	0.0	0	0.0
**33**	19	1.4	17	1.3	1	1.6	0	0.0	1	50.0
**35**	5	0.4	5	0.4	0	0.0	0	0.0	0	0.0
**39**	33	2.4	26	2.0	2	3.3	5	17.9	0	0.0
**40**	5	0.4	5	0.4	0	0.0	0	0.0	0	0.0
**42**	43	3.1	36	2.8	4	6.6	3	10.7	0	0.0
**43**	3	0.2	2	0.2	1	1.6	0	0.0	0	0.0
**44**	19	1.4	16	1.2	1	1.6	2	7.1	0	0.0
**45**	29	2.1	26	2.0	3	4.9	0	0.0	0	0.0
**51**	31	2.2	21	1.6	4	6.6	6	21.4	0	0.0
**52**	24	1.7	17	1.3	3	4.9	4	14.3	0	0.0
**53**	33	2.4	25	1.9	7	11.5	1	3.6	0	0.0
**54**	27	1.9	20	1.5	6	9.8	1	3.6	0	0.0
**56**	39	2.8	31	2.4	4	6.6	4	14.3	0	0.0
**58**	14	1.0	11	0.8	3	4.9	0	0.0	0	0.0
**59**	13	0.9	9	0.7	3	4.9	1	3.6	0	0.0
**61**	9	0.6	9	0.7	0	0.0	0	0.0	0	0.0
**62**	2	0.1	2	0.2	0	0.0	0	0.0	0	0.0
**66**	7	0.5	6	0.5	0	0.0	1	3.6	0	0.0
**67**	3	0.2	3	0.2	0	0.0	0	0.0	0	0.0
**68**	20	1.4	16	1.2	1	1.6	3	10.7	0	0.0
**70**	13	0.9	12	0.9	0	0.0	1	3.6	0	0.0
**72**	1	0.1	0	0.0	0	0.0	1	3.6	0	0.0
**73**	7	0.5	7	0.5	0	0.0	0	0.0	0	0.0
**74**	5	0.4	4	0.3	1	1.6	0	0.0	0	0.0
**81**	6	0.4	4	0.3	1	1.6	1	3.6	0	0.0
**82**	12	0.9	9	0.7	1	1.6	2	7.1	0	0.0
**90**	2	0.1	2	0.2	0	0.0	0	0.0	0	0.0
**114**	1	0.1	1	0.1	0	0.0	0	0.0	0	0.0

## Discussion

This study provides basic information about the HPV type-specific prevalence in the Czech Republic before the onset of routine vaccination and allows for further surveillance of changes in the prevalence of a number of HPV genotypes. The prevalence has been studied in the screening population of Czech women. Two methods for HPV detection were used. In order to provide internationally comparable data, the most widely used commercially available set HR HC2 was selected. The second method, on the other hand, allows for a very sensitive detection of a wide spectrum of HPV types and also of multiple infections. The two methods yielded concordant results in 91% of samples. HPV and HR HPV are highly prevalent in the Czech population. The highest prevalence was observed in the age group of 21-25 years. In women older than 55 years, no multiple infection and almost no LR types were detected. The majority of the women had normal cytological findings, only 6.5% of the women had abnormal cytological findings. This is in agreement with the annual data in the Czech biggest cytological laboratory which performs about 600 000 cytological examinations of screening cytology per year. Approximately 7.5% of women from the screening population were infected with the vaccinal HR types and 6.5% by closely and distantly related HR types while LR vaccinal types were much less frequent (1.4%). Similarly to other countries, the most prevalent HR type was HPV 16 but other HR HPV types (HPV 56, 39/45, 18, and 51) that were the most commonly detected in the Czech population were different from those reported for European countries by Bruni et al. (HPV 16, 31, 18, 39, 33, and 66) [5]. 

Meta-analyses as well as a pooled analysis of HPV type-specific prevalence in women with normal cytological findings worldwide have been conducted [4,5,23] but the previously published data from the Czech Republic [6] were not included because of their unsuitability. Data on HPV type-specific prevalence in the screening population from Eastern Europe are still limited. None of the Eastern European countries was included in the first pooled analyses [23]. In the meta-analysis of de Sanjosé et al. only data from Russia were analyzed while Bruni et al. included also data from Poland, Hungary, Belarus and Latvia [4,4,5]. However, for some countries, e.g. Hungary, only the total prevalence of HR and LR types was assessed, for other countries, a limited number of HPV types were analyzed or only a part of the HPV positive samples were typed. Recently, data from Slovenia have also been published [24]. 

The worldwide HPV prevalence in women with normal cytology in the pooled analysis was 10.4%, the HPV prevalence in Europe was 8.1%, and the distribution varied substantially between continents. Eastern Europe was represented by only one study from Russia with 309 women enrolled. HPV prevalence in this study was much higher than in other European regions (29.1%). HPV 16 was the most prevalent type worldwide (2.5%) as well as in Eastern Europe (7.4%), but this result needs to be considered with caution since only 100 HPV positive samples were typed in the Russian study. The prevalence of HPV 16 in other European regions was much lower (1.6% to 3.0%) [4]. 

The meta-analysis of one million women with normal cytological findings [5] found an overall prevalence of HPV of 11.7%, comparably worldwide. This meta-analysis included data from five East European countries and confirmed a substantially higher prevalence of HPV in these five countries in comparison with other European regions (21.4% vs. 8.8%-10%). In our study, the prevalence of any HPV type in women with normal cytological findings was 25.6% and was higher than in Poland (16.6%). The prevalences of HR HPV types in women with normal cytological findings were lower in Poland and Slovenia (9.6% and 10.7%, respectively) than in the Czech Republic (19.7% and. 15.6% by BS-RLB and HR HC2, respectively). In the screening population from Russia, Belarus and Latvia, the prevalence of HR HPV types was 27.2%. Since in the four studies, women from the screening population were enrolled, the differences in the prevalence can reflect the real differences between the populations of the four East European countries or can be attributed to the fact that different methods were used for HPV detection as shown before [5]. The Polish study used the GP5+/6+ PCR system which is less sensitive than the modified system used in our study. Similar system to ours was applied by Schmitt et al. [25] to the screening population of Belgian women and the prevalence obtained in their study were comparable to our data. In the Slovenian study, a system which targets less HPV types was utilized. However, in the study of Kulmala et al. [26], a system which detects only nine HR HPV types was used and the reported prevalence was comparably high as in the Czech population. Another source of variability is the distinction between the HR and LR HPV types. In the analyses, we have included both the HR and pHR HPV types as HR types (see materials and methods). If we used the same definition of HR types as the Polish study [27], the HR HPV prevalences in the Czech population would be still much higher, 20.4% for the whole cohort and 17.9% for women with normal cytological findings. Our data confirm the exceptionally high prevalence of HPV in the screening population of East European countries, and are also comparable to the findings of numerous other studies performed around the world with the most widely utilized set HC2 (for review see 5). 

In the screening population of the Czech women, the highest prevalence of HPV was found in the age group of 21-25 years. The highest prevalence in the same age category was found in both less and more developed countries. In Eastern European countries, the peak was observed in the age group of 25-34 years [5]. HPV type-specific data in the meta-analysis of 1 million women were available for less than one fourth of subjects. HPV 16 was the most prevalent type worldwide (3.2%), followed by HPV 18, 52, 31, and 58. The type-specific prevalence separately for East European countries was not evaluated in this meta-analysis. Of five studies from East European countries included in the meta-analysis and additionally the study from Slovenia, only two provide type-specific prevalence in the screening population of women [24,27]. HPV 16 was the most prevalent type in all three populations of women with normal cytological findings (Czech, Polish, and Slovenian) (4.8%, 2.8%, and 2.3%, respectively). The higher prevalence of HPV 16 in the Czech population is most likely due to the very high sensitivity (1fg/reaction) of our system for HPV 16 detection. The prevalence of other types varied between studies, stressing the importance of surveillance of HPV infection on a national level. In the Czech Republic, the second most prevalent HR type, in agreement with the Polish study, was HPV 56 (2.4% vs. 1.8%), followed by HPV 39, 45, 18, and 51 (2.0-1.6%). In Slovenia, HPV 31 was detected as the second most common type (2.5%) in agreement with the results of meta-analyses (2.3% for the European Continent). Of the LR types, in agreement with the Polish study, the most prevalent was HPV 42 (2.8% in the Czech Republic and 2.3% in Poland). In the Czech Republic, the next most prevalent LR types were HPV 54, 6, and 44 (1.9-1.4%) unlike in the Polish study (HPV 83, JC9710 (cand90), 70, and 53). Some of the discrepancies can be related to the sensitivity of BS-RLB for the particular types, e.g. our system has a “lower” specificity for the detection of HPV 31 and 59 (1pg/reaction) while for most remaining types, the sensitivity is much higher (1-10 fg/reaction) (personal observation). In women with normal cytological findings, the prevalence of the HR vaccinal types 16/18 was 6.6%, the prevalences of the closely and distantly related HR types were 2.8% and 2.9%. LR vaccinal types were detected in 1.5% of women, but only until 40 years of age. For all vaccinal types, the highest prevalence was detected in the age group of 21-25 years (12.2% for HPV 16/18; 3.3% for HPV 6/11). This observation is in agreement with our data from the prevalence study in the population of sexually active women in the Czech Republic who undergo HPV vaccination. About 12% of these women were infected with the vaccinal types. As both vaccines show decreased or null effectiveness in women infected with vaccinal HPV types, a proper understanding by the physicians of the possible limitations of the efficiency of HPV vaccines in these women is very important. Physicians should also be able to transfer the information to patients in order to minimize their psychological harm and the media attempts to discredit HPV vaccines. 

In our previous study, HPV type-specific prevalence rates were assessed in patients with SCC and CIN 1-3 and type-specific relative risk (RR) was calculated but comparative data for the normal population of women were not available. In the present study, we have further compared the screening population of women with patients with SCC [28] for all seven HR HPV types detected previously in Czech women with SCC. A RR of 26.6 (95% CI=17.1-41.5) was observed for HPV 16. Similarly, significantly increased prevalence rates were observed for HPV 18, 31, 33, and 45 (RR=3.3-6.1). This means that healthy women infected with HPV 16 have 55% higher risk of developing SCC than HPV negative women. 

In conclusion, this is the first large study of HPV-type specific prevalence in the Czech Republic on a female screening population, and the third study in an East European country. Two methods for HPV detection were used in order to provide internationally comparable data. Our study showed a very high prevalence of HPV, and this high prevalence correlates with the consistently high incidence of cervical cancer in the Czech Republic. The highest prevalence was observed in the age group of 21-25 years, but also in women older than 31 years the prevalence of HR HPV was more than 15%. As in other studies, HPV 16 was the most common HPV type. However, the other HR HPV types that were commonly detected in the Czech population were different from those reported in European countries before.

The highest prevalence of vaccinal HPV 16/18 types was detected in the age group of 21-25 years. This group of women currently undergoes vaccination on personal request without reimbursement. Routine HPV detection is not recommended in the Czech Republic prior to vaccination and therefore physicians should inform the patients about the decreased effectiveness of vaccination if the woman is infected at the time of vaccination by the vaccinal types of HPV. This study provides baseline information about the HPV type-specific prevalence in the Czech Republic before the onset of routine vaccination and allows for further surveillance of changes in the prevalence of a number of HPV genotypes.
